# Hyperuricemia as a Biomarker for Circadian Syndrome: A Cross-Sectional Study

**DOI:** 10.3390/clockssleep8020033

**Published:** 2026-06-04

**Authors:** Joud AlBashtawi, Hana Zeidan, Abdulrahman Al-Tairi, Najlaa Al-Marri, Mohammed Abdel Hamid, Reem Odaiba, Shaikha Al-Kurbi, Ramez Jaradat, Susu M. Zughaier, Habib H. Farooqui

**Affiliations:** 1 College of Medicine, QU Health, Qatar University, Doha P.O. Box 2713, Qatar; 2 Department of Basic Medical Sciences, College of Medicine, QU Health, Qatar University, Doha P.O. Box 2713, Qatar; szughaier@qu.edu.qa; 3 Department of Population Medicine, College of Medicine, QU Health, Qatar University, Doha P.O. Box 2713, Qatar

**Keywords:** circadian syndrome, hyperuricemia, metabolic syndrome

## Abstract

Circadian Syndrome (CircS) links metabolic, behavioral, and mental health challenges to disrupted circadian rhythm. Hyperuricemia (HUA) is associated with cardiometabolic disorders; however, its connection to CircS remains incompletely understood. This study aims to determine if HUA can serve as a biomarker for CircS. We analyzed the National Health and Nutrition Examination Survey (NHANES) data (2005–2020) from 27,410 adults. CircS was defined as the presence of at least five out of eight components, encompassing the traditional metabolic syndrome criteria plus three additional factors: short sleep duration, depression, and non-alcoholic fatty liver disease (NAFLD). HUA was set at serum uric acid levels >6 mg/dL (357 µmol/L) for females and >7 mg/dL (416 µmol/L) for males. Multivariable logistic regression identified predictors of CircS, and dose–response relationships were explored. Of 27,410 participants, 2076 (7.57%) had CircS. HUA was found to be the strongest independent predictor of CircS, with an odds ratio (OR) of 3.26 (95% uncertainty interval (UI) 2.80–3.80, *p* < 0.001). Other important risk factors included chronic kidney disease (CKD) (OR 2.80), female sex (OR 1.44), and smoking (OR 1.29). In addition, older age, lower educational level, and lower income were also linked with higher risk. Higher uric acid levels were consistently linked to metabolic components of CircS more strongly than to depression and short sleep. In conclusion, HUA is linked with a greater risk of CircS. Although testing is simple and widely available, it could be used for early risk detection. Future studies should determine if lowering uric acid improves circadian health.

## 1. Introduction

Circadian Syndrome (CircS) is an emerging clinical construct that extends beyond the traditional framework of Metabolic Syndrome (MetS) by integrating behavioral and neuropsychological dimensions associated with circadian rhythm disruption [1]. It is defined by the presence of at least five out of the following eight criteria: elevated triglycerides, reduced high-density lipoprotein cholesterol (HDL-C), non-alcoholic fatty liver disease (NAFLD), increased waist circumference, elevated fasting blood glucose, high blood pressure, short sleep duration, and depressive symptoms [2]. While MetS primarily addresses metabolic risk, CircS includes a broader perspective by including sleep and mood disturbances [1,3,4]. This growing understanding implies that the body’s circadian rhythm affects metabolic functions, hormones, and cardiac functions [5,6]. Therefore, it highlights the need for a more holistic framework in preventing and managing cardiometabolic diseases [3,7].

Hyperuricemia (HUA) is characterized by serum uric acid level greater than 6 mg/dL (357 µmol/L) in females and 7 mg/dL (416 µmol/L) in males [8]. In addition to being a byproduct of purine metabolism, uric acid has been implicated in several pathophysiological mechanisms that intersect with insulin resistance, type 2 diabetes, and hypertension [9]. HUA is increasingly regarded as a mediator of metabolic risk, often preceding MetS components, and its reduction may mitigate cardiometabolic risk [9,10]. However, the role of HUA within broader metabolic constructs such as CircS remains unclear, representing the gap that this study seeks to fill.

CircS and HUA are prevalent in the general population [11]. Although the prevalence of CircS is still being established, recent population-based studies have reported rates ranging from 31.5% in China to 32.9% in the United States [12,13]. On the other hand, HUA is estimated to affect approximately 21% of adults in the United States [6].

Clarifying whether hyperuricemia is associated with the development or progression of CircS represents an important gap with significant clinical relevance. Given the modifiable nature of serum uric acid through both lifestyle changes and pharmacological agents such as xanthine oxidase inhibitors [14,15], the potential for improving cardiometabolic health outcomes is vast. Exploring this relationship using predictive modeling could also clarify shared pathophysiological pathways between metabolic dysfunction and circadian disruption, paving the way for more integrated and personalized prevention strategies [3]. Hence, this study aims to assess whether HUA can serve as a predictor of CircS.

## 2. Results

### 2.1. Baseline Characteristics

Table 1 presents the baseline demographic, socioeconomic, and clinical characteristics of the study population, stratified by CircS status. Of the 27,410 participants, 2076 (7.57%) met CircS criteria. Participants with CircS were older (median 60.0 vs. 42.0 years, *p* < 0.001) and more often female (55.2% vs. 47.8%, *p* < 0.001). CircS prevalence was higher in the lowest income category (PIR < 1: 27.2% vs. 21.4%, *p* < 0.001) and among those with lower educational attainment. HUA was more prevalent in the CircS group (40.0% vs. 16.7%, *p* < 0.001). The prevalence of individual CircS components among participants is presented in the Supplementary Material (See Supplementary Figure S1).

Comparison of included and excluded participants demonstrated several statistically significant differences (See Supplementary Table S1). Excluded individuals were older (median age 52 vs. 44 years, *p* < 0.001) and more likely to be female. They also had lower educational attainment and lower income levels. Differences were observed across ethnic groups, although the distributions remained broadly comparable. In addition, excluded participants had a higher prevalence of hyperuricemia (45.2% vs. 16.7%) and chronic kidney disease (4.3% vs. 2.8%).

### 2.2. Primary Analysis

In the logistic regression model, HUA was the strongest independent predictor of CircS, with a 3.26-fold increase in odds, compared to normal serum uric acid levels (OR: 3.26; 95% UI: 2.80–3.80; *p* < 0.001). CKD also showed a strong association (OR: 2.80; 95% UI: 2.06–3.81; *p* < 0.001). Females showed increased odds compared to males (OR: 1.44; 95% UI: 1.24–1.68; *p* < 0.001). Age, modeled using restricted cubic splines, had a nonlinear effect: risk rose from young adulthood to midlife, then plateaued and slightly declined at older ages (see Supplementary Figure S2). Using Hispanic participants as the reference group, Black (OR: 0.49; 95% UI: 0.39–0.62; *p* < 0.001) and Other/Multiracial participants (OR: 0.53; 95% UI: 0.38–0.72; *p* < 0.001) demonstrated significantly lower odds of CircS, while no significant difference was observed among White participants.

Among lifestyle factors, alcohol intake was associated with reduced odds (OR: 0.59; 95% UI: 0.48–0.73; *p* < 0.001), while smoking was associated with increased odds (OR: 1.28; 95% UI: 1.10–1.50; *p* < 0.001). Graduate-level education lowered odds (OR: 0.54; 95% UI: 0.41–0.71; *p* < 0.001), whereas high school education showed no significant difference (OR: 1.03; *p* = 0.785). Socioeconomic status, measured by PIR, showed a gradient: participants with PIR ≥ 5 had lower odds (OR: 0.47; 95% UI: 0.36–0.61; *p* < 0.001), as did PIR 4–4.9 (OR: 0.56; 95% UI: 0.41–0.76; *p* < 0.001) with smaller, but significant, reductions for PIR 1–3.9. Diet quality was not significantly associated with CircS risk. Higher physical activity was linked to lower odds (OR: 0.99; 95% UI: 0.99–0.99; *p* = 0.010), despite the small per-unit estimate. To visually demonstrate the multifactorial nature of CircS, a forest plot was generated to display the predictive ORs from the logistic regression model (see Supplementary Table S2, Figure S3). The full multivariable logistic regression model is presented in Table 2.

### 2.3. Secondary Analysis

#### 2.3.1. Model Performance

The predictive performance of the final model was evaluated using the ROC curve and the Linktest. The model demonstrated good discrimination, with an area under the ROC curve (AUC) of 0.79, indicating a strong ability to distinguish between participants with and without CircS (see Supplementary Figure S4). Model specification was supported by the Linktest, showing that predicted values were significantly associated with the outcome while their squared terms were not, suggesting no evidence of misspecification (See Supplementary Table S3). Together, these results confirm the adequacy and robustness of the final predictive model.

#### 2.3.2. Dose–Response Analysis with Individual Components:

**Figure 1:**
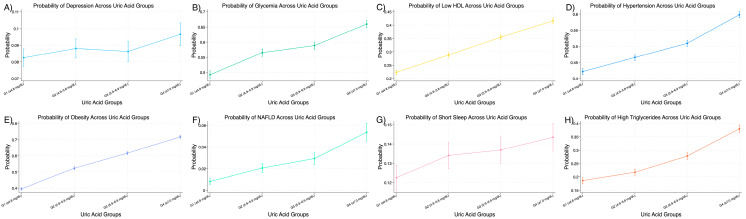
Probability of individual components of Circadian Syndrome (CircS) across serum uric acid Groups (G1–G4). Panels (**A**–**H**) illustrate the estimated probability of (**A**) depression, (**B**) glycemia, (**C**) low HDL cholesterol, (**D**) hypertension, (**E**) obesity, (**F**) non-alcoholic fatty liver disease (NAFLD), (**G**) short sleep duration, and (**H**) high triglycerides across increasing uric acid groups (e.g., G1–G4). Data are presented as predicted probabilities with error bars representing 95% confidence intervals. Uric acid groups were categorized into quartiles based on serum uric acid levels. The total sample size was *n* = 27,410, with group-specific sample sizes provided where applicable. Abbreviations: HDL, high-density lipoprotein; NAFLD, non-alcoholic fatty liver disease.

To explore the dose–response relationship between uric acid levels and the individual components of CircS, participants were categorized into 4 groups using quartiles based on serum uric acid concentration: G1 (≤4.8 mg/dL (≤286 µmol/L)), G2 (4.9–5.8 mg/dL (292–345 µmol/L)), G3 (5.9–6.9 mg/dL (351–410 µmol/L)), and G4 (≥7.0 mg/dL (≥416 µmol/L))(see Figure 1).

Visual inspection of the probabilities across uric acid groups revealed a consistent upward trend for several CircS components. Groups with higher uric acid levels were associated with progressively greater probability of hypertension (from ~0.42 in G1 to ~0.6 in G4), hyperglycemia (~0.47 to ~0.68), hypertriglyceridemia (~0.18 to ~0.38), low HDL cholesterol (~0.23 to ~0.41), obesity (~0.4 to ~0.72), and NAFLD (~0.01 to ~0.05). These findings suggest the relationship of progressive increase in uric acid levels and the development of different CircS components.

For short sleep duration, a modest and less consistent upward trend was observed with relatively wide uncertainty intervals, indicating potential variability or instability in the relationship. Similarly, the association with depressive symptoms appeared weak, with overlapping uncertainty intervals and minimal separation across groups.

In addition to analyzing uric acid levels by quartiles, dose–response relationships were also assessed using uric acid as a continuous variable (see Supplementary Figure S5*)*. Both approaches showed consistent positive associations with individual components of CircS, reinforcing a graded and robust relationship. Full regression outputs for these analyses are presented in Supplementary Tables S4–S19.

To assess the robustness of the association between HUA and CircS, a sensitivity analysis was conducted using three alternative definitions of elevated uric acid levels based on varying sex-specific thresholds: Model A (>7.0 mg/dL (416 µmol/L) for males, >6.0 mg/dL (357 µmol/L) for females), Model B (>6.8 mg/dL (405 µmol/L) for males, >5.7 mg/dL (339 µmol/L) for females), and Model C (>7.5 mg/dL (446 µmol/L) for males, >6.5 mg/dL (387 µmol/L) for females). A forest plot displaying the results yielded consistently elevated ORs, ranging from 3 to 3.5 approximately (see Supplementary Figure S6). This consistency across multiple thresholds confirms the stability and robustness of the predictive association between uric acid levels and CircS. Full regression outputs for the three models are provided in Supplementary Tables S20–S22.

In the 10% random subsample analysis, hyperuricemia remained significantly associated with circadian syndrome (OR 3.03, 95% CI 1.73–5.31), with effect estimates comparable to the primary analysis. Model diagnostics demonstrated acceptable discrimination and no evidence of misspecification (Supplementary Tables S23–S25, Figure S7).

## 3. Discussion

This study supports that hyperuricemia is a significant predictor of CircS, extending prior evidence beyond MetS by incorporating behavioral, neuropsychological, and clinical factors, and highlighting its potential utility as a predictive biomarker.

Our study contributes to the growing evidence on the association between CircS and HUA, while offering several distinct strengths. Mao et al. (2025) reported a significant association between CircS and HUA (OR = 1.23; 95% CI: 1.07–1.40) using a 7-component CircS definition [16]. In comparison, we used an 8-component CircS definition by including NAFLD, which adds metabolic relevance. The robustness of our findings was supported by comprehensive dose–response, sensitivity, and subsample validation analyses. Our results are consistent with Zhang et al. (2025), who found that short sleep (OR = 1.10) and daytime napping (OR = 1.34) were associated with HUA [17]. Findings from Raya-Cano et al. (2022) and Darmawan et al. (2017) also support the link between HUA, metabolic syndrome, and NAFLD [18,19]. In contrast, Wang et al. (2018) reported a negative association between serum uric acid and depression, which our study did not assess [9]. These differences highlight the importance of expanding CircS definitions and tailoring research to regional and demographic contexts to better capture early metabolic risk.

HUA is increasingly recognized as a potential biomarker of CircS, reflecting underlying circadian–metabolic interactions rather than direct measures of circadian disruption. Previous studies suggest that elevated uric acid promotes oxidative stress, inflammasome activation (e.g., NLRP3), and endothelial dysfunction, contributing to cardiometabolic disorders and symptom clustering in CircS [20,21]. Uric acid also interacts with circadian regulation: monosodium urate crystals disrupt immune cell clock function, while serum uric acid shows diurnal variation regulated by circadian genes controlling renal transporters [22,23]. Short sleep is further linked to oxidative stress and HUA [24,25], which may reflect a bidirectional relationship contributing to both circadian and metabolic dysregulation [26,27]. Collectively, these mechanisms support HUA as a plausible biomarker of CircS [6,28]. However, as this study was conducted within a predictive modeling framework, which prioritizes predictive performance over causal inference, caution is required when interpreting potential biological mechanisms underlying these associations.

The stronger association between HUA and CircS in females may reflect sex-specific differences in uric acid metabolism. Estrogen promotes renal uric acid clearance, and its decline after menopause can elevate serum levels, compounding risks of metabolic and circadian disruption [29,30]. Notably, the elevated risk observed in individuals with normal BMI suggests HUA may act as an early metabolic warning sign, which is consistent with the metabolically unhealthy normal weight (MUNW) phenotype [30,31]. This underscores the value of HUA for early risk stratification beyond traditional anthropometric measures.

Several predictors emerged as significant risk factors of CircS, underscoring its multifaceted nature. Advancing age was strongly linked to higher odds of CircS, consistent with aging-related attenuation of circadian rhythms and associated metabolic and sleep disturbances [32,33]. Higher odds in females may reflect hormonal influences on metabolism and sleep, as well as differences in the expression of CircS components, such as depressive symptoms [34]. Conversely, lower odds among Black and multiracial participants compared with Hispanic individuals suggest the influence of genetic, environmental, and social determinants of health. These differences may reflect variations in dietary habits, sleep patterns, socioeconomic conditions, healthcare access, and other unmeasured factors. Although our analysis adjusted for key confounders, residual confounding cannot be excluded and warrants further investigation.

CKD was a strong independent predictor of CircS, reflecting the close link between renal, metabolic, and circadian functions. CKD has been associated with chronic metabolic dysfunction, including HUA, and may also be linked to circadian disruption through shared regulatory pathways [35,36]. Smoking was also associated with higher odds of CircS, consistent with its role in driving inflammation, oxidative stress, and circadian disruption [37,38]. Alcohol intake was linked to lower odds, though this may reflect underreporting, measurement limitations, or behavioral changes following diagnosis.

Socioeconomic status, indicated by education and PIR, showed a favorable gradient with higher levels linked to lower odds of CircS. This highlights the impact of social determinants on health [39,40]. Higher education and economic stability often correlate with better health literacy, access to nutritious food, promotion of physical activity, reduced stress, improved sleep, and supporting metabolic and circadian homeostasis.

The lack of association between the Healthy Eating Index (HEI-2015) and CircS may reflect limitations in measurement rather than the absence of effect. Specifically, the HEI captures dietary quality but does not account for circadian-relevant behaviors such as meal timing and chrono-nutrition, which may influence circadian and metabolic regulation.

The findings have important public health and clinical implications. HUA emerges as a potential biomarker for CircS with utility in early risk stratification, provided that these results are validated in future prospective and clinical studies [41,42]. Its widespread availability and modifiability through lifestyle or pharmacologic interventions make it a low-cost tool to identify at-risk patients. The multifactorial nature of CircS, reflected in demographic, socioeconomic, lifestyle, and clinical predictors, underscores the need for multidimensional interventions, including addressing social determinants, promoting sleep hygiene, managing stress, and encouraging balanced diets [43,44].

### 3.1. Limitations

A substantial proportion of participants were excluded due to missing data. Importantly, excluded individuals differed from those included in several key demographic and clinical characteristics, including age, socioeconomic status, and prevalence of hyperuricemia and CKD. These findings suggest that missingness may not be completely at random and could introduce selection bias. However, the consistency of the observed associations across multiple sensitivity analyses supports the robustness of the study findings.

Several methodological limitations should also be acknowledged. First, the cross-sectional design precludes causal inference, and the reliance on single-time-point measurements of uric acid and CircS components may not fully capture long-term patterns. In addition, several variables, including sleep, alcohol consumption, dietary intake, and physical activity, -were assessed using self-reported questionnaires. Although commonly used in epidemiological research, these measures are subject to recall and social desirability bias, which may lead to misclassification and attenuation of effect estimates. In particular, the alcohol intake variable, defined using fixed daily thresholds, may not adequately capture important consumption patterns such as binge drinking or distinguish between never, former, and current drinkers, thereby reducing measurement precision.

Similarly, physical activity was assessed using self-reported data, which may underestimate its true physiological impact. Future studies incorporating objective measures, such as actigraphy, are warranted to better characterize the relationship between physical activity and CircS. A key limitation is that depression was assessed using the PHQ-9, which, while validated for symptom severity, is not a diagnostic tool and does not capture functional impairment; therefore, clinically diagnosed depression cannot be confirmed. Cirrhosis was initially considered as a covariate but was excluded from the final analyses due to substantial missing data.

Finally, the absence of objective circadian markers, such as melatonin levels, actigraphy, or light exposure data, as well as genetic and hormonal measures, limits our ability to directly assess circadian rhythm disruption. Consequently, the observed associations may partly reflect broader metabolic dysfunction rather than circadian-specific effects. Additionally, unmeasured confounders, including chronotype, shift work, and genetic variation in circadian-related pathways, may have influenced the observed relationships.

To ensure appropriate interpretation of our findings, circadian-related claims should be approached with caution. Although Circadian Syndrome (CircS) incorporates behavioral and psychological components linked to circadian rhythm disturbances, the NHANES dataset lacks objective circadian measures. Therefore, the associations observed in this study should be interpreted as reflecting relationships with circadian–metabolic syndrome indicators rather than direct evidence of circadian disruption. Furthermore, the cross-sectional design limits causal inference and precludes mechanistic conclusions. Nevertheless, the robustness of our results is supported by multiple sensitivity analyses, dose–response assessments, and subsample validation. Future research should employ prospective and longitudinal designs incorporating objective circadian phenotyping, such as actigraphy, melatonin and cortisol profiling, light exposure measurements, and chronotype assessments, to elucidate causal pathways and underlying biological mechanisms.

### 3.2. Strength

This study has several notable strengths. First, it utilized a large, nationally representative sample from the National Health and Nutrition Examination Survey (NHANES), enhancing the generalizability of the findings to the U.S. adult population. Second, the comprehensive evaluation of demographic, socioeconomic, lifestyle, and clinical variables allowed for rigorous multivariable adjustment and minimized potential confounding. Third, the use of standardized laboratory measurements for serum uric acid ensured high data reliability and reproducibility. Fourth, the study incorporated a multidimensional definition of Circadian Syndrome (CircS), integrating metabolic, behavioral, and psychological components to provide a holistic assessment of circadian–metabolic health. Additionally, the robustness of the findings was reinforced through multiple analytical approaches, including dose–response analyses, sensitivity analyses using alternative hyperuricemia thresholds, and validation using a 10% random subsample. Finally, by evaluating hyperuricemia within the broader CircS framework, this study offers novel insights into the interplay between metabolic and circadian health and supports the potential utility of serum uric acid as a clinically accessible biomarker for early risk stratification.

## 4. Materials and Methods

### 4.1. Study Design and Sample Selection

This study utilized publicly available data from the National Health and Nutrition Examination Survey (NHANES), National Center for Health Statistics, Centers for Disease Control and Prevention (CDC), Hyattsville, MD, USA. NHANES, is a cross-sectional survey designed to assess the health and nutritional status of people living in the United States [45]. Data is gathered through interviews, comprehensive questionnaires, laboratory testing, and physical assessments [2]. The study is ethically overseen by the National Center for Health Statistics, Institutional Ethics Review Board, and all participants provide written informed consent. Detailed information about the survey’s data and procedures can be found at https://wwwn.cdc.gov/nchs/nhanes/Default.aspx (accessed on 20 January 2025).

This cross-sectional study utilized data from seven consecutive NHANES cycles (2005–2020), initially comprising 76,496 participants. Individuals under 18 years of age (*n* = 30,516) were excluded, as were those with missing data on serum uric acid (*n* = 28,577) or CircS status (*n* = 18,570). After applying exclusion criteria, a total of 27,410 participants were retained for analysis.

To assess potential selection bias arising from missing data, we compared baseline characteristics between included and excluded participants.

### 4.2. Predictors

The primary outcome analysis incorporated the following variables as Predictors.

#### 4.2.1. Uric Acid Levels

Uric acid was quantified from serum samples using enzymatic colorimetric assays, a standard clinical laboratory method [46]. Patients with HUA were defined by having a serum uric acid level greater than 6 mg/dL (357 µmol/L) in females and 7 mg/dL (416 µmol/L) in males [47].

#### 4.2.2. Demographics

Demographic information was obtained through self-report, including gender, age (years), ethnicity (Hispanic, white, black, and others/multi-racial), educational level, and socioeconomic status. Socioeconomic status was assessed using the level of education and the poverty income ratio (PIR), which was calculated by dividing a family’s income by the appropriate family poverty threshold that corresponds to the family’s size, year, and state. PIR was then grouped into six categories, with higher values reflecting greater economic stability.

#### 4.2.3. Lifestyle Factors & Clinical Predictors

Diet quality was assessed using the Healthy Eating Index–2015 (HEI-2015), which measures adherence to the 2015–2020 Dietary Guidelines for Americans across 13 components. Total scores range from 0 to 100, with higher scores indicating better diet quality. Participants were classified as having healthy (HEI ≥ 67.6), moderate (HEI 51.2–67.5), or poor (HEI ≤ 51.1) diet quality, based on thresholds derived from NHANES percentile distributions [48].

Physical activity was measured using the Global Physical Activity Questionnaire (GPAQ), which captures activity across occupational, transport, and recreational domains. Activities were assigned MET values: 8 METs for vigorous-intensity and 4 METs for moderate-intensity or transport-related activity. Total weekly MET-hours were calculated accordingly [49,50].

Smoking status was determined via self-reported responses to NHANES questionnaires. Alcohol consumption was categorized as low-risk or at-risk, using sex-specific thresholds: men reporting > 4 drinks/day and women > 3 drinks/day were classified as at-risk drinkers [51]. Chronic Kidney Disease (CKD) status was derived from self-reported physician diagnosis.

### 4.3. Outcome Measure: Circadian Syndrome Status

CircS was defined by the presence of at least five out of eight diagnostic components [2]. These included: (1) depressive symptoms, identified by a Patient Health Questionnaire (PHQ-9) score of ≥10, indicating moderate to severe depression [52]; (2) short sleep duration, defined as self-reported sleep of less than 6 h per night [53], and (3) non-alcoholic fatty liver disease (NAFLD), which was assessed using the United States Fatty Liver Index (US-FLI) [54] with a score of ≥30 considered diagnostic in the absence of other liver disease etiologies, such as heavy alcohol consumption (>4 standard drinks/day (56 g ethanol/day) for men and >3 (42 g/day) for women) [51], hepatitis B infection (HBsAg positive), or hepatitis C infection (HCV RNA positive). The remaining five components correspond to the standard criteria for Metabolic Syndrome (MetS) [55]: (4) elevated waist circumference (≥102 cm in men and ≥88 cm in women); (5) elevated blood pressure (systolic ≥130 mm Hg and/or diastolic ≥85 mm Hg) or current use of antihypertensive medication; (6) low HDL cholesterol (<1 mmol/L (40 mg/dL) in men and <1.3 mmol/L (50 mg/dL) in women) or use of medication for low HDL-C; (7) elevated triglycerides (≥150 mg/dL(1.7 mmol/L)) or use of medication for hypertriglyceridemia; and (8) elevated fasting plasma glucose (≥100 mg/dL (5.6 mmol/L)), use of glucose-lowering medication, or a diagnosis of diabetes or prediabetes.

### 4.4. Statistical Analyses

The primary aim is to evaluate whether HUA can serve as a predictor of CircS. Hence, we employed a predictive modeling approach using logistic regression. Prediction modeling emphasizes assessing predictive accuracy rather than causal inference, making it appropriate for assessing the utility of HUA as a potential biomarker in clinical risk stratification

Categorical variables were summarized as frequencies (*n*) and percentages (%). For continuous variables, normality was assessed using histograms; however, all were found to be non-normally distributed; therefore, they were reported as medians with interquartile ranges (IQR). Group comparisons were conducted using Pearson’s chi-squared test for categorical variables and the Wilcoxon rank-sum test for continuous variables. Data was visualized using bar graphs and line graphs as appropriate.

To account for potential nonlinearity in the relationship between age and outcomes, age was modeled using restricted cubic splines with four knots, following recommended practices in regression modeling strategies [56]. This flexible approach captures complex associations without imposing a strict linear or quadratic form. All multivariable models were adjusted for age using these spline terms. For simplicity and clarity, age was modeled as a linear covariate in visual presentations, such as forest plots.

To evaluate the extent to which the study data diverged from the tested hypothesis (a population model assuming no effect), *p*-values were calculated. A smaller *p*-value indicated greater statistical divergence from the tested hypothesis. Results with *p*-values below the predefined threshold of 0.05 were described as statistically divergent, suggesting that random error alone may not adequately explain the observed findings. In addition to *p*-values, point estimates and their corresponding 95% uncertainty intervals (UIs) were reported. The 95% UI reflects the range of test hypotheses for which the observed data lie within the central 95% of their predicted distribution, representing models that remain plausible explanations for the data [57].

To assess predictive performance, a Receiver Operating Characteristic (ROC) curve was generated, and the area under the curve (AUC) was computed. Model specification was evaluated using the Linktest. For dose–response exploration, the relationship between serum uric acid and individual components of CircS was examined by modeling uric acid both as a continuous variable and in quartiles. Quartile cutoffs were defined based on the sample distribution. A sensitivity analysis was conducted using three alternative definitions of HUA, applying varying sex-specific serum uric acid thresholds to test the robustness of associations. All statistical analyses were performed using Stata version SE18.5 (Stata Corp., College Station, TX, USA).

Finally, the robustness of the primary findings was examined using a 10% random subsample of the study population, which was analyzed using the same multivariable logistic regression model. Results of this validation analysis are presented in the Supplementary Material (Supplementary Tables S23–S25, Figure S7).

## 5. Conclusions

HUA was independently associated with CircS, highlighting its relationship with circadian-related indicators. Its accessibility and modifiability make it a potential biomarker for early risk stratification. Future research should investigate the causal relationship and evaluate whether interventions targeting uric acid levels influence circadian and metabolic health outcomes in longitudinal or experimental settings.

## Figures and Tables

**Table 1:** Baseline demographic, socioeconomic, lifestyle, and clinical characteristics of the study population stratified by Circadian Syndrome status *(n =* 27,410).

Factor	Level	No Circadian Syndrome *n =* 25,334 *n (%)*	Circadian Syndrome *n =* 2076 *n (%)*	*p*-Value
Age in years, median (IQR)		42.0 (28.0, 59.0)	60.0 (49.0, 68.0)	<0.001
Sex				
	Male	13,221 (52.2%)	930 (44.8%)	<0.001
	Female	12,113 (47.8%)	1146 (55.2%)	
Ethnicity				
	Hispanic	6395 (25.2%)	557 (26.8%)	<0.001
	White	10,178 (40.2%)	989 (47.6%)	
	Black	5531 (21.8%)	381 (18.4%)	
	Others	3230 (12.7%)	149 (7.2%)	
Poverty Income Ratio (PIR)				
	PIR < 1	4934 (21.4%)	515 (27.2%)	<0.001
	PIR 1–1.9	5784 (25.0%)	610 (32.3%)	
	PIR 2–2.9	3509 (15.2%)	261 (13.8%)	
	PIR 3–3.9	2663 (11.5%)	179 (9.5%)	
	PIR 4–4.9	1856 (8.0%)	109 (5.8%)	
	PIR ≥ 5	4360 (18.9%)	217 (11.5%)	
Education Level				
	Below high school	5172 (22.1%)	702 (33.9%)	<0.001
	Highschool	12,080 (51.6%)	1110 (53.7%)	
	Graduate	6172 (26.3%)	256 (12.4%)	
Hyperuricemia (HUA)				
	No hyperuricemia	21,098 (83.3%)	1245 (60.0%)	<0.001
	Hyperuricemia	4236 (16.7%)	831 (40.0%)	
Diet Quality				
	Healthy	1366 (5.4%)	107 (5.2%)	0.53
	Moderate	5540 (21.9%)	435 (21.0%)	
	Poor	18,428 (72.7%)	1534 (73.9%)	
Smoking Status				
	Non-Smoker	12,222 (58.3%)	765 (44.4%)	<0.001
	Smoker	8736 (41.7%)	957 (55.6%)	
Alcohol consumption				
	Low risk drinking	12,918 (76.9%)	939 (84.2%)	<0.001
	At-risk drinking	3877 (23.1%)	176 (15.8%)	
Physical Activity, median (IQR)		16.0 (0.0, 68.0)	4.0 (0.0, 32.0)	<0.001
Chronic Kidney Disease (CKD) Status				
	Healthy	22,901 (97.8%)	1868 (90.5%)	<0.001
	CKD	512 (2.2%)	196 (9.5%)	

**Table 2:** Multivariable logistic regression model predicting Circadian Syndrome (CircS) using hyperuricemia and other demographic, socioeconomic, lifestyle, and clinical predictors (*n* = 13,782).

CircS		Odds Ratio (OR)		*p*-Value	95% UI	
Hyperuricemia (HUA) *		3.26		<0.001	2.80–3.80	
Age (splines) **						
Spline term 1		1.14		<0.001	1.10–1.18	
Spline term 2		0.88		0.009	0.80–0.97	
Spline term 3		1.13		0.336	0.88–1.45	
Sex						
Male		REF				
Female		1.44		<0.001	1.24–1.68	
Ethnicity						
Hispanic		REF				
White		0.93		0.477	0.77–1.13	
Black		0.49		<0.001	0.39–0.62	
Others		0.53		<0.001	0.38–0.72	
Smoking						
Non-Smoker		REF				
Smoker		1.28		0.001	1.10–1.50	
Alcohol						
Low risk drinking		REF				
At-risk drinking		0.59		<0.001	0.48–0.73	
Education						
Below High School		REF				
High School		1.03		0.785	0.85–1.24	
Graduate		0.54		<0.001	0.41–0.71	
Poverty Income Ratio (PIR)						
PIR < 1		REF				
PIR 1–1.9		0.79		0.030	0.65–0.98	
PIR 2–2.9		0.61		<0.001	0.48–0.79	
PIR 3–3.9		0.71		0.009	0.54–0.92	
PIR 4–4.9		0.56		<0.001	0.41–0.76	
PIR ≥ 5		0.47		<0.001	0.36–0.61	
CKD Status						
No CKD		REF				
CKD		2.80	<0.001			2.06–3.81
Diet quality						
High		REF				
Moderate		0.73	0.123			0.49–1.09
Poor		1.04	0.837			0.72–1.51
Physical Activity		0.99	0.010			0.99–0.99
_cons		0.00	<0.001			0.001–0.002

Model fit: McFadden’s pseudo-R^2^ = 0.152. Discrimination: ROC AUC = 0.790. Specification (linktest): _hat significant (*p* < 0.001); _hatsq not significant (*p* = 0.335). * Hyperuricemia was defined as having serum uric acid levels >7 mg/dL (357 µmol/L) in males and >6 mg/dL (416 µmol/L) in females. ** Age was modeled using restricted cubic splines with 4 knots to allow for nonlinear effects. Spline terms represent different segments of the age distribution.

## Data Availability

Publicly available datasets were analyzed in this study. These data can be found in the National Health and Nutrition Examination Survey (NHANES) repository, hosted by the Centers for Disease Control and Prevention (CDC): https://wwwn.cdc.gov/nchs/nhanes/ (accessed on 20 January 2025).

## Supplementary Materials

The following supporting information can be downloaded at: https://www.mdpi.com/article/10.3390/clockssleep8020033/s1.

## Abbreviations

The following abbreviations are used in this manuscript:

AUC	Area Under the Curve
BMI	Body Mass Index
CDC	Centers for Disease Control and Prevention
CI	Confidence Interval
CircS	Circadian Syndrome
CKD	Chronic Kidney Disease
HDL-C	High-Density Lipoprotein Cholesterol
HEI-2015	Healthy Eating Index–2015
HUA	Hyperuricemia
HCV	Hepatitis C Virus
HBsAg	Hepatitis B Surface Antigen
IQR	Interquartile Range
MET	Metabolic Equivalent of Task
MetS	Metabolic Syndrome
MUNW	Metabolically Unhealthy Normal Weight
NAFLD	Non-Alcoholic Fatty Liver Disease
NCHS	National Center for Health Statistics
NHANES	National Health and Nutrition Examination Survey
OR	Odds Ratio
PHQ-9	Patient Health Questionnaire-9
PIR	Poverty Income Ratio
ROC	Receiver Operating Characteristic
UI	Uncertainty Interval
US-FLI	United States Fatty Liver Index
