# Supplementary material for: Hyperuricemia as a Biomarker for Circadian Syndrome: A Cross-Sectional Study
Source: Clocks Sleep. 2026 Jun 4;8(2):33. doi: 10.3390/clockssleep8020033 (PMC13297815; doi:10.3390/clockssleep8020033)
Supplement: Supplementary file 1 [file clockssleep-08-00033-s001.zip › clockssleep-4191569-supplementary.pdf]

Hyperuricemia as a Biomarker for Circadian syndrome: A Cross-Sectional Study

(Supplementary Material)

Supplementary Figure 1. ***Circadian Syndrome (CircS) Components Prevalence***

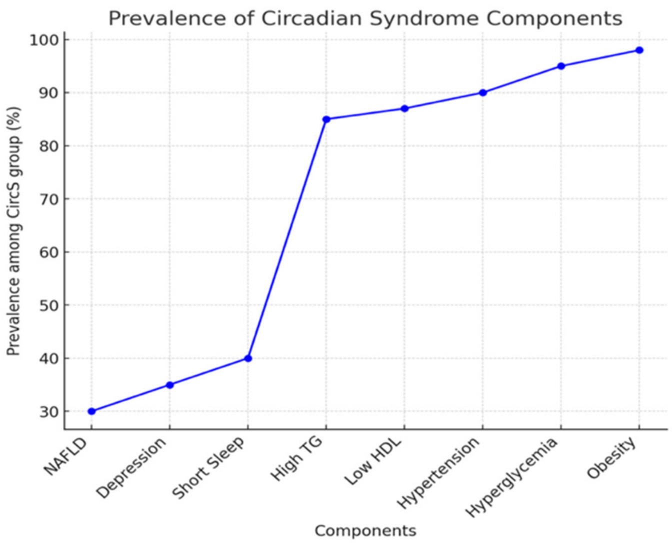

Supplementary Table 1. ***Comparison of Baseline Characteristics Between Included and Excluded Participants***

Variable	Included	Excluded	p-value
**Age, median (IQR)**	44 (32)	52 (31)	<0.001
**Sex**			<0.001
Male	51.6%	44.4%	
Female	48.4%	55.6%	
**Ethnicity**			<0.001
Hispanic	25.5%	25.0%	
White	41.0%	39.4%	
Black	21.4%	24.7%	
Others	12.2%	10.8%	
**Education**			<0.001
Below high school	23.0%	27.1%	
High school	51.7%	53.6%	
Graduate	25.3%	19.3%	
**PIR**			<0.001
PIR <1	21.7%	23.3%	
PIR ≥5	18.4%	15.6%	

**Hyperuricemia**			<0.001
Yes	16.7%	45.2%	
**CKD**			<0.001
Yes	2.8%	4.3%	

**Variable**	**Included**	**Excluded**	**p-value**
**Age, median (IQR)**	44 (32)	52 (31)	<0.001
**Sex**			<0.001
Male	51.6%	44.4%	
Female	48.4%	55.6%	
**Ethnicity**			<0.001
Hispanic	25.5%	25.0%	
White	41.0%	39.4%	
Black	21.4%	24.7%	
Others	12.2%	10.8%	
**Education**			<0.001
Below high school	23.0%	27.1%	
High school	51.7%	53.6%	
Graduate	25.3%	19.3%	
**PIR**			<0.001
PIR <1	21.7%	23.3%	
PIR ≥5	18.4%	15.6%	
**Hyperuricemia**			<0.001
Yes	16.7%	45.2%	
**CKD**			<0.001
Yes	2.8%	4.3%	

Supplementary Table 2. Logistic Regression Model for Circadian Syndrome Using Age as a Continuous Covariate (Model Used for Forest Plot Visualization)

**Variable**	**Categories**	**Odds Ratio**	**P-value**	**95% UI**
**Hyperuricemia**		3.16	<0.001	2.71 – 3.67
**Age (in years)**		1.03	<0.001	1.03 – 1.04
**Sex**				

	Female	1.48	<0.001	1.28 – 1.72
**Race**				
	White	0.82	0.038	0.68 – 0.99
	Black	0.51	<0.001	0.41 – 0.64
	Others	0.52	<0.001	0.38 – 0.71
**Smoking**				
	Smoker	1.36	<0.001	1.17– 1.58
**alcohol**				
	At risk drinking	0.62	<0.001	0.50 – 0.76
**Education**				
	High school	1.02	0.814	0.85 – 1.23
	Graduate	0.54	<0.001	0.42 – 0.71
**Poverty-to-income ratio (PIR)**				
	PIR 1–1.9	0.78	0.016	0.63 – 0.95
	PIR 2–2.9	0.60	<0.001	0.46 – 0.76
	PIR 3–3.9	0.72	0.012	0.55 – 0.93
	PIR 4–4.9	0.58	0.001	0.43 – 0.80
	PIR ≥5	0.54	<0.001	0.42 – 0.70
**Chronic kidney disease (CKD)**				
	CKD	2.79	<0.001	2.05 – 3.79
**Healthy eating index (HEI)**				
	Moderate	0.73	0.032	0.55 – 0.98
	Poor	0.76	0.116	0.54 – 1.05
**Physical activity**		1.00	0.992	0.84 – 1.19

**_cons**	0.02	<0.001	0.01 – 0.02

Supplementary figure 2. ***Impact of Age on the Odds of Circadian Syndrome***

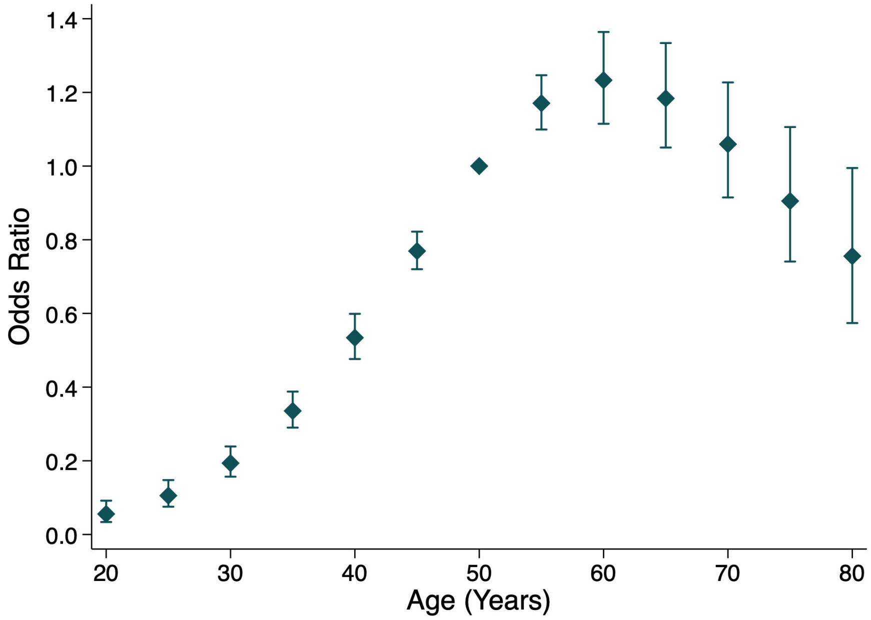

Supplementary figure 3. ***Forest plot***

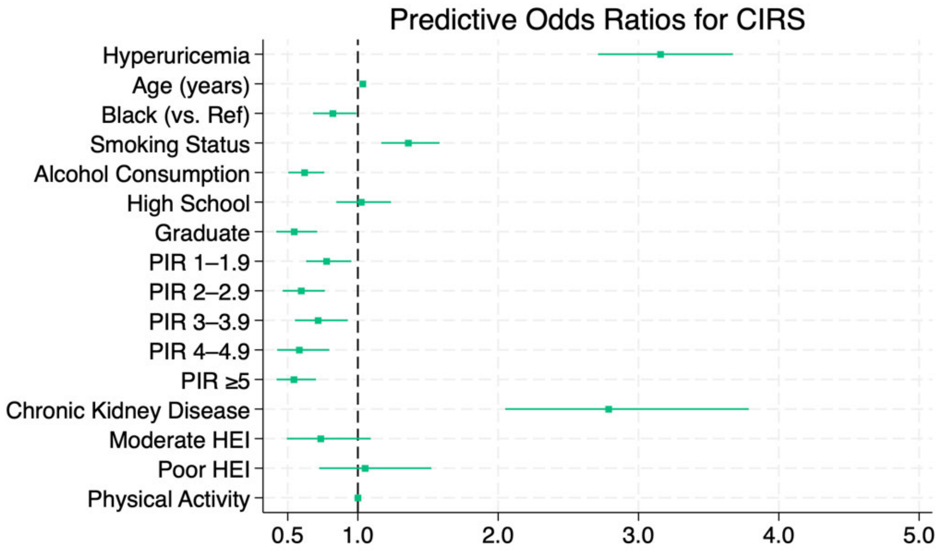

Supplementary figure 4. ***ROC curve***

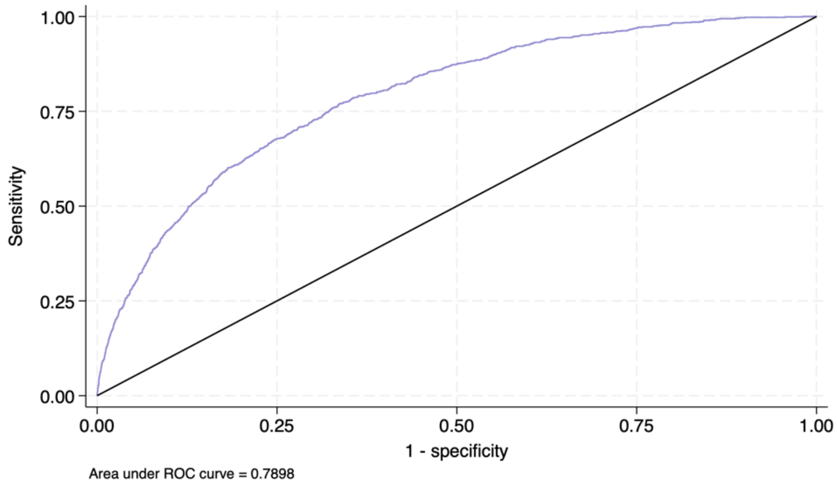

Supplementary Table 3. ***Linktest results***

Variable	Coefficient	Std. Err.	z	P-Value	95% UI
**_hat**	1.105	0.115	9.57	<0.001	0.88 – 1.33
**_hatsq**	0.022	0.023	0.96	0.335	-0.02 – 0.07
**_cons**	0.102	0.138	0.74	0.459	-0.17 – 0.37

Dose response relationship of uric acid with individual components of CircS:

1. ***Uric acid as quartiles:***

Supplementary Table 4. ***Uric acid and hypertension***

Variable	Odds ratio	P-Value	95% UI

**Uric acid quartiles**			
**G2 (4.9–5.8 mg/dL)**	1.32	<0.001	1.22 – 1.42
**G3 (5.9–6.9 mg/dL)**	1.73	<0.001	1.59 – 1.87
**G4 (≥7.0 mg/dL)**	3.03	<0.001	2.78 – 3.30
**Age (Splines)**			
**age_spline1**	1.09	<0.001	1.08 – 1.10
**age_spline2**	1.02	0.282	0.99 – 1.05
**age_spline3**	0.92	0.042	0.85 – 1.00
**Gender**			
**Female**	1.04	0.186	0.98 – 1.11
**Ethnicity**			
**White**	1.00	0.909	0.93 – 1.07
**Black**	2.23	<0.001	2.06 – 2.42
**Others/multi-racial**	1.05	0.385	0.95– 1.16
**_cons**	0.02	<0.001	0.01 – 0.10

Supplementary Table 5. Uric acid and Glycemia

Variable	Odds ratio	P-Value	95% UI
**Uric acid quartiles**			
**G2 (4.9–5.8 mg/dL)**	1.43	<0.001	1.31 – 1.55
**G3 (5.9–6.9 mg/dL)**	1.60	<0.001	1.48– 1.75
**G4 (≥7.0 mg/dL)**	2.32	<0.001	2.12 – 2.54
**Age (Splines)**			
**age_spline1**	1.06	<0.001	1.04 – 1.07
**age_spline2**	1.04	0.014	1.01– 1.07

**age_spline3**	0.83	<0.001	0.76 – 0.89
**Gender**			
**Female**	0.68	<0.001	0.64 – 0.73
**Ethnicity**			
**White**	0.57	<0.001	0.53– 0.61
**Black**	0.65	<0.001	0.59 – 0.70
**Others/multi-racial**	0.81	<0.001	0.73 – 0.89
**_cons**	0.10	<0.001	0.08 – 0.13

***Supplementary Table 6**. Uric acid and dyslipidemia (HDL)*

Variable	Odds ratio	P-Value	95% UI
**Uric acid quartiles**			
**G2 (4.9–5.8 mg/dL)**	1.42	<0.001	1.33– 1.51
**G3 (5.9–6.9 mg/dL)**	1.95	<0.001	1.83– 2.08
**G4 (≥7.0 mg/dL)**	2.56	<0.001	2.39 – 2.74
**Age (splines)**			
**age_spline1**	1.02	<0.001	1.01 – 1.03
**age_spline2**	0.94	<0.001	0.92– 0.93
**age_spline3**	1.13	<0.001	1.07– 1.20
**Gender**			
**Female**	1.79	<0.001	1.71 – 1.88
**Ethnicity**			
**White**	0.76	<0.001	0.72– 0.80
**Black**	0.50	<0.001	0.47 – 0.53
**race2: Others/Multi-racial**	0.64	<0.001	0.60 – 0.69

**_cons**	0.17	<0.001	0.14 – 0.21

Supplementary Table 7. Uric acid and dyslipidemia (TGs)

Variable	Odds ratio	P-value	95% UI
**Uric acid quartiles**			
**G2 (4.9–5.8 mg/dL)**	1.22	<0.001	1.11 – 1.36
**G3 (5.9–6.9 mg/dL)**	1.73	<0.001	1.56 – 1.91
**G4 (≥7.0 mg/dL)**	2.83	<0.001	2.55 – 3.13
**Age (splines)**			
**age_spline1**	1.06	<0.001	1.05 – 1.07
**age_spline2**	0.92	<0.001	0.89 – 0.95
**age_spline3**	1.14	0.004	1.043 – 1.25
**Gender**			
**Female**	0.94	0.085	0.87 – 1.01
**Ethnicity**			
**White**	0.82	<0.001	0.75 – 0.88
**Black**	0.27	<0.001	0.24 – 0.30
**Others/multi-racial**	0.68	<0.001	0.61 – 0.76
**_cons**	0.05	<0.001	0.03– 0.06

Supplementary Table 8. Uric acid and obesity

Variable	Odds ratio	P-Value	95% UI
**Uric acid quartiles**			
**G2 (4.9–5.8 mg/dL)**	1.93	<0.001	1.81 – 2.05
**G3 (5.9–6.9 mg/dL)**	3.12	<0.001	2.91 – 3.34

**G4 (≥7.0 mg/dL)**	5.50	<0.001	5.10 – 5.93
**Age (splines)**			
**age_spline1**	1.07	<0.001	1.06 – 1.07
**age_spline2**	0.92	<0.001	0.90– 0.94
**age_spline3**	1.14	<0.001	1.08 – 1.21
**Gender**			
**Female**	5.90	<0.001	5.59– 6.24
**Ethnicity**			
**White**	0.85	<0.001	0.80 – 0.90
**Black**	0.91	0.004	0.85 – 0.97
**Others/multi-racial**	0.29	<0.001	0.27 – 0.32
**_cons**	0.03	<0.001	0.02 – 0.03

***Supplementary Table 9**. Uric acid and NAFLD*

Variable	Odds ratio	P-value	95% UI
**Uric acid quartiles**			
**G2 (4.9–5.8 mg/dL)**	2.60	<0.001	1.76 – 3.83
**G3 (5.9–6.9 mg/dL)**	3.74	<0.001	2.55 – 5.50
**G4 (≥7.0 mg/dL)**	7.08	<0.001	4.85 – 10.34
**Age (splines)**			
**age_spline1**	1.07	<0.001	1.03 – 1.11
**age_spline2**	0.94	0.282	0.86 – 1.05
**age_spline3**	1.04	0.794	0.78 – 1.38
**Gender**			
**Female**	1.17	0.156	0.94 – 1.46

**Ethnicity**			
**White**	0.91	0.424	0.72 – 1.15
**Black**	0.39	<0.001	0.29 – 0.54
**Others/multi-racial**	0.70	0.050	0.50– 1.00
**_cons**	0.00	<0.001	0.00 – 0.00

Supplementary Table 10. Uric acid and Depression

Variable	Odds ratio	P-value	95% UI
**Uric acid quartiles**			
**G2 (4.9–5.8 mg/dL)**	1.07	0.158	0.97 – 1.19
**G3 (5.9–6.9 mg/dL)**	1.05	0.367	0.94 – 1.17
**G4 (≥7.0 mg/dL)**	1.19	0.002	1.06 – 1.33
**Age (splines)**			
**age_spline1**	1.01	0.319	0.99– 1.02
**age_spline2**	1.04	0.039	1.00 – 1.07
**age_spline3**	0.83	<0.001	0.75 – 0.91
**Gender**			
**Female**	1.84	<0.001	1.69 – 1.99
**Ethnicity**			
**White**	0.91	0.046	0.83 – 0.99
**Black**	0.92	0.107	0.83 – 1.02
**Others/multi-racial**	0.66	<0.001	0.57 – 0.76
**_cons**	0.05	<0.001	0.04 – 0.07

Supplementary Table 11. Uric acid and short sleep

Variable	Odds ratio	P-value	95% UI
**Uric acid quartiles**			
**G2 (4.9–5.8 mg/dL)**	1.11	0.014	1.02 – 1.21
**G3 (5.9–6.9 mg/dL)**	1.12	0.004	1.04 – 1.24
**G4 (≥7.0 mg/dL)**	1.20	<0.001	1.10 – 1.32
**Age (splines)**			
**age_spline1**	1.04	<0.001	1.03 – 1.05
**age_spline2**	0.92	<0.001	0.90 – 0.95
**age_spline3**	1.18	<0.001	1.09 – 1.27
**Gender**			
**Female**	0.98	0.567	0.92 – 1.05
**Ethnicity**			
**White**	0.88	0.002	0.82 – 0.96
**Black**	1.95	<0.001	1.80 – 2.11
**Others/multi-racial**	0.90	0.071	0.81 – 1.01
**_cons**	0.04	<0.001	0.03 – 0.05

2. ***Uric acid as continuous variable:***

Supplementary Figure 5. Probability of individual CircS components across uric acid levels (mg/dL)

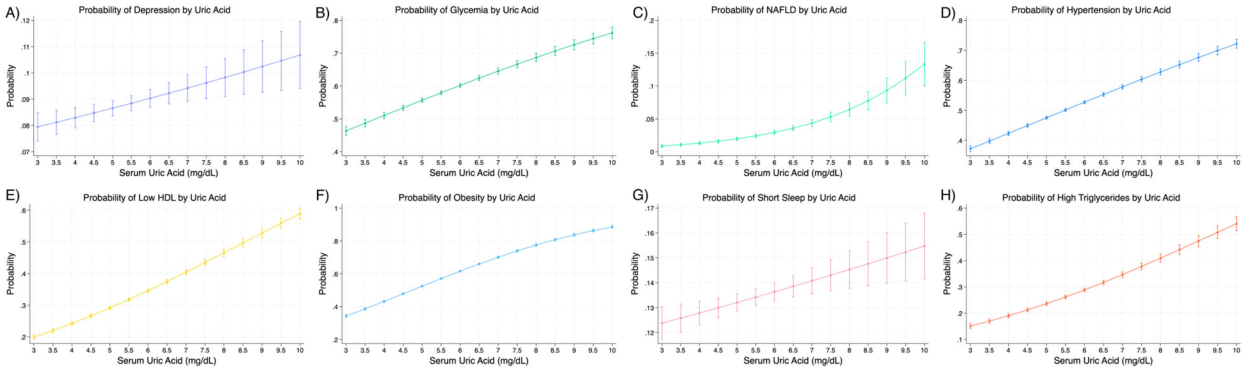

Supplementary Table 12. Uric acid and hypertension

Variable	Odds ratio	P-Value	95% UI
**Uric acid (mg/dL)**	1.38	<0.001	1.35 – 1.41
**Age (Splines)**			
**age_spline1**	1.09	<0.001	1.08 – 1.10
**age_spline2**	1.01	0.320	0.99 – 1.04
**age_spline3**	0.92	0.049	0.85 – 0.99
**Gender**			
**Female**	1.09	0.009	1.02 – 1.15
**Ethnicity**			
**White**	0.99	0.826	0.93 – 1.06
**Black**	2.21	<0.001	2.03 – 2.39
**Others/multi-racial**	1.03	0.512	0.93 – 1.14
**_cons**	0.00	<0.001	0.00 – 0.00

Supplementary Table 13. Uric acid and glycemia

Variable	Odds ratio	P-Value	[95% conf. interval]
**Uric acid (mg/dL)**	1.25	<0.001	1.22 – 1.28
**Age (splines)**			
**age_spline1**	1.06	<0.001	1.05 – 1.07
**age_spline2**	1.04	0.013	1.01 – 1.07
**age_spline3**	0.82	<0.001	0.76 – 0.89
**Gender**			
**Female**	0.69	<0.001	0.65 – 0.74
**Ethnicity**			
**White**	0.56	<0.001	0.52 – 0.61
**Black**	0.64	<0.001	0.58 – 0.69
**Others/multi-racial**	0.80	<0.001	0.72 – 0.89
**_cons**	0.04	<0.001	0.03 – 0.059

Supplementary Table 14. Uric acid and dyslipidemia (HDL)

Variable	Odds ratio	P-Value	95% UI
**Uric acid (mg/dL)**	1.30	<0.001	1.28 – 1.32
**Age (splines)**			
**age_spline1**	1.02	<0.001	1.01 – 1.03
**age_spline2**	0.94	<0.001	0.92 – 0.96
**age_spline3**	1.13	<0.001	1.07– 1.19
**Gender**			
**Female**	1.81	<0.001	1.72– 1.90
**Ethnicity**			

**White**	0.75	<0.001	0.71 – 0.80
**Black**	0.49	<0.001	0.46 – 0.52
**Others/multi-racial**	0.64	<0.001	0.59 – 0.69
**_cons**	0.07	<0.001	0.06 – 0.08

Supplementary Table 15. Uric acid and dyslipidemia (TGs)

Variable	Odds ratio	P-Value	95% UI
**Uric acid (mg/dL)**	1.33	<0.001	1.30 – 1.36
**Age (splines)**			
**age_spline1**	1.06	<0.001	1.05 – 1.07
**age_spline2**	0.92	<0.001	0.89 – 0.95
**age_spline3**	1.14	0.005	1.04 – 1.24
**Gender**			
**Female**	0.94	0.111	0.88 – 1.01
**Ethnicity**			
**White**	0.81	<0.001	0.75 – 0.88
**Black**	0.26	<0.001	0.24 – 0.29
**Others/multi-racial**	0.67	<0.001	0.60 – 0.75
**_cons**	0.02	<0.001	0.01 – 0.02

Supplementary Table 16. Uric acid and obesity

Variable	Odds ratio	P-Value	95% UI
**Uric acid (mg/dL)**	1.60	<0.001	1.57 – 1.64

**Age (splines)**			
**age_spline1**	1.07	<0.001	1.06 – 1.07
**age_spline2**	0.92	<0.001	0.90 – 0.94
**age_spline3**	1.14	<0.001	1.07 – 1.21
**Gender**			
**Female**	5.98	<0.001	5.66 – 6.31
**Ethnicity**			
**White**	0.84	<0.001	0.80 – 0.90
**Black**	0.89	0.001	0.84 – 0.95
**Others/multi-racial**	0.28	<0.001	0.26 – 0.31
**_cons**	0.01	<0.001	0.00 – 0.01

Supplementary Table 17. Uric acid and NAFLD

Variable	Odds ratio	P-Value	95% UI
**Uric acid (mg/dL)**	1.52	<0.001	1.42 – 1.63
**Age (splines)**			
**age_spline1**	1.07	<0.001	1.03 – 1.11
**age_spline2**	0.95	0.337	0.85 – 1.06
**age_spline3**	1.02	0.896	0.77 – 1.35
**Gender**			
**Female**	1.13	0.289	0.90 – 1.40
**Ethnicity**			
**White**	0.93	0.562	0.74 – 1.18
**Black**	0.38	<0.001	0.28 – 0.53
**Others/multi-racial**	0.73	0.076	0.51 – 1.03

**_cons**	0.00	<0.001	0.00 – 0.00

Supplementary Table 18. Uric acid and depression

Variable	Odds ratio	P-Value	95% Ui
**Uric acid (mg/dL)**	1.05	0.001	1.02 – 1.08
**Age (splines)**			
**age_spline1**	1.01	0.314	0.99 – 1.02
**age_spline2**	1.04	0.042	1.00 – 1.07
**age_spline3**	0.83	<0.001	0.75 – 0.91
**Gender**			
**Female**	1.85	<0.001	1.71– 2.01
**Ethnicity**			
**White**	0.91	0.043	0.83 – 1.00
**Black**	0.92	0.096	0.83 – 1.02
**Others/multi-racial**	0.66	<0.001	0.57 – 0.76
**_cons**	0.05	<0.001	0.03 – 0.06

Supplementary Table 19. Uric acid and short sleep

Variable	Odds ratio	P-Value	95% UI
**Uric acid (mg/dL)**	1.04	0.001	1.02 – 1.06
**Age (splines)**			
**age_spline1**	1.04	<0.001	1.03 – 1.05
**age_spline2**	0.93	<0.001	0.90 – 0.95

**age_spline3**	1.17	<0.001	1.09 – 1.27
**Gender**			
**Female**	0.97	0.343	0.91 – 1.03
**Ethnicity**			
**White**	0.90	0.002	0.82 - 0.96
**Black**	1.95	<0.001	1.81 - 2.11
**Others/multi-racial**	0.91	0.075	0.81 - 1.01
**_cons**	0.04	<0.001	0.03 - 0.05

Sensitivity analysis:

Supplementary Table 20. ***Model A (>7mg/dL in males, >6mg/dL in females)***

Variable	Odds ratio	P-Value	95% UI
**Hyperuricemia (Model A)**	3.21	<0.001	2.75 – 3.75
**Age (Splines)**			
**age_spline1**	1.14	<0.001	1.10 – 1.18
**age_spline2**	0.87	0.006	0.80 – 0.96
**age_spline3**	1.16	0.244	0.90 – 1.49
**Gender**	1.47	<0.001	1.27 – 1.70
**Ethnicity**	0.76	<0.001	0.70 – 0.83
**smoking**	1.36	<0.001	1.17 – 1.59
**alcohol**	0.59	<0.001	0.48 – 0.72
**Education**	0.78	<0.001	0.69 – 0.88
**Poverty- to- income ratio (PIR)**	0.88	<0.001	0.84 – 0.92

**Chronic kidney disease (CKD)**	2.81	<0.001	2.07 – 3.83
**Healthy eating index (HEI)**	1.23	0.004	1.07 – 1.42
**Physical activity**	1.00	0.015	1.00 – 1.00
**_cons**	0.00	<0.001	0.00 – 0.00

Supplementary Table 21. Model B (>6.8mg/dL in males, >5.7mg/dL in females)

Variable	Odds ratio	P-Value	95% UI
**Hyperuricemia (model B)**	3.15	<0.001	2.72 –3.66
**age_spline1**	1.14	<0.001	1.10 – 1.18
**age_spline2**	0.87	0.006	0.79 – 0.96
**age_spline3**	1.16	0.229	0.91 – 1.49
**Gender**	1.467	<0.001	1.26 –1.70
**Ethnicity**	0.76	<0.001	0.69 – 0.82
**smoking**	1.37	<0.001	1.18 – 1.60
**alcohol**	0.58	<0.001	0.47 – 0.72
**Education**	0.78	<0.001	0.69 – 0.89
**Poverty- to- income ratio (PIR)**	0.87	<0.001	0.83 – 0.91
**Chronic kidney disease (CKD)**	2.88	<0.001	2.12 – 3.91
**Healthy eating index (HEI)**	1.24	0.003	1.08 – 1.43
**Physical activity**	1.00	0.016	1.00 – 1.00
**_cons**	0.00	<0.001	0.00 – 0.00

Supplementary Table 22. Model C (>7.5mg/dL in males, >6.5mg/dL in females)

Variable	Odds ratio	P-Value	95% UI
**Hyperuricemia (Model C)**	3.53	<0.001	2.97– 4.18
**age_spline1**	1.14	<0.001	1.01– 1.18
**age_spline2**	0.87	0.006	0.79– 0.96
**age_spline3**	1.16	0.231	0.91– 1.49
**Gender**	1.46	<0.001	1.26 – 1.69
**Ethnicity**	0.76	<0.001	0.70 – 0.83
**smoking**	1.37	<0.001	1.18 – 1.59
**alcohol**	0.60	<0.001	0.49 – 0.73
**Education**	0.80	<0.001	0.70 – 0.89
**Poverty- to- income ratio (PIR)**	0.88	<0.001	0.84 – 0.92
**Chronic kidney disease (CKD)**	2.83	<0.001	2.08 – 3.85
**Healthy eating index (HEI)**	1.22	0.005	1.06 – 1.41
**Physical activity**	1.00	0.016	1.00 – 1.00
**_cons**	0.00	<0.001	0.00 – 0.00

Supplementary Figure 6. Visualization of sensitivity analysis results

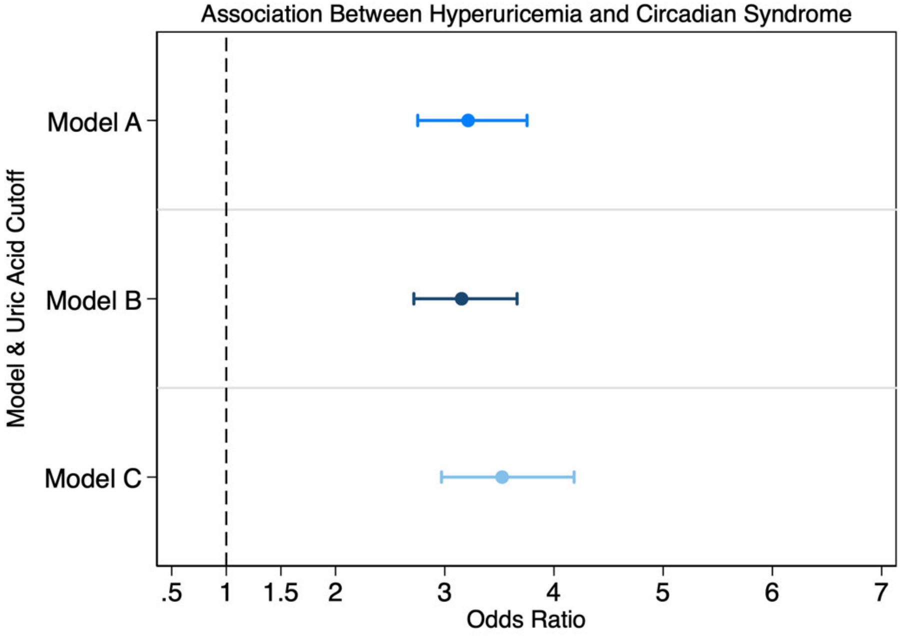

10% Random Subsample Validation Analysis

A 10% random subsample of the study population (n = 2,733) was analyzed using the same multivariable logistic regression model as the primary analysis to assess robustness.

***Supplementary Table 23.** Baseline distribution in 10% random subsample*

	No Hyperuricemia	Hyperuricemia	Total
No Circadian Syndrome	2,113	416	2,529
Circadian Syndrome	137	67	204
Total	2,250	483	2,733

***Supplementary Table 24.** Multivariable logistic regression model in 10% subsample*

Variable	Odds Ratio (OR)	P-value	95% CI
Hyperuricemia	3.03	<0.001	1.73 – 5.31
Age spline 1	1.11	0.138	0.97 – 1.27

Age spline 2	1.05	0.782	0.73 – 1.51
Age spline 3	0.60	0.280	0.24 – 1.52
Female (vs Male)	1.89	0.016	1.13 – 3.18
White	1.01	0.969	0.54 – 1.88
Black	0.38	0.021	0.17 – 0.86
Others / Multiracial	0.29	0.060	0.08 – 1.06
Smoking	1.95	0.018	1.12 – 3.39
Alcohol	0.73	0.352	0.37 – 1.42
High school education	0.71	0.305	0.37 – 1.36
Graduate education	0.35	0.017	0.15 – 0.83
CKD	5.11	0.015	1.38 – 18.96
HEI – Moderate	1.69	0.540	0.31 – 9.14
HEI – Poor	2.69	0.232	0.53 – 13.67
Physical activity	1.00	0.244	1.00 – 1.00

***Supplementary Figure 7.* ROC curve for 10% subsample model**

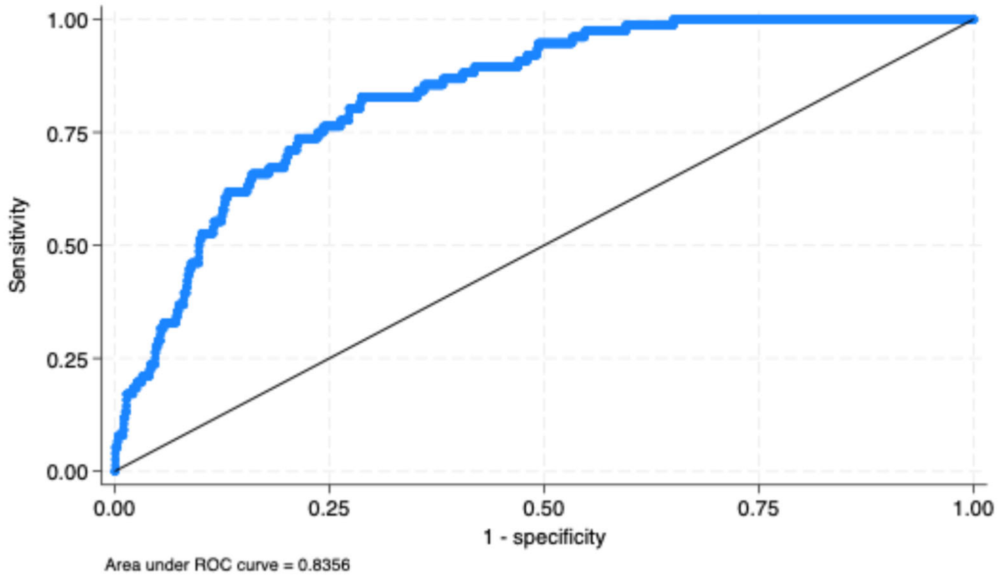

Supplementary Table 25. Link test for model specification

Variable	Coefficient	Std. Error	z	P-value	95% CI
_hat	0.6015	0.3588	1.68	0.094	−0.1017 – 1.3047
_hatsq	−0.0848	0.0746	−1.14	0.256	−0.2311 – 0.0614
Constant	−0.3649	0.4140	−0.88	0.378	−1.1763 – 0.4465
